# Modulation of CXCL-8 expression in human melanoma cells regulates tumor growth, angiogenesis, invasion, and metastasis

**DOI:** 10.1002/cam4.28

**Published:** 2012-10-04

**Authors:** Sheng Wu, Seema Singh, Michelle L Varney, Scott Kindle, Rakesh K Singh

**Affiliations:** 1 Department of Pathology and Microbiology, University of Nebraska Medical CenterOmaha, Nebraska; 2 Department of Oncologic Sciences, Mitchell Cancer Institute, University of South AlabamaMobile, Alabama

**Keywords:** Angiogenesis, CXCL-8, melanoma, metastasis, proliferation

## Abstract

CXCL-8, a chemokine secreted by melanoma and stromal cells, serves as a growth and angiogenic factor for melanoma progression. This study evaluated how modulation of CXCL-8 levels in melanoma cell lines with different tumorigenic and metastatic potentials affected multiple tumor phenotypes. A375P cells (CXCL-8 low expressor) were stably transfected with a CXCL-8 mammalian expression vector to overexpress CXCL-8, whereas A375SM cells (CXCL-8 high expressor) were transfected with a CXCL-8 antisense expression vector to suppress CXCL-8 expression. Subsequent cell proliferation, migration, invasion, and soft-agar colony formation were analyzed, and in vivo tumor growth and metastasis were evaluated using mouse xenograft models. Our data demonstrate that overexpression of CXCL-8 significantly enhanced primary tumor growth and lung metastasis, accompanied by increased microvessel density in vivo, as compared with vector control-transfected cells. We also observed increased clonogenic ability, growth, and invasive potential of CXCL-8 overexpressing cells in vitro. Knockdown of CXCL-8 using an antisense vector resulted in increased cell death and reduced tumor growth relative to control. Taken together, these data confirm that CXCL-8 expression plays a critical role in regulating multiple cellular phenotypes associated with melanoma growth and metastasis.

## Introduction

The last 80 years have seen an estimated 30-fold increase in the lifetime risk of developing melanoma [1]. For this deadly skin disease, the fifth most common cancer among American men and the seventh among American women [2], limited therapies are currently offered that prolong survival, even though a variety of targets have been discovered that tumors depend on for sustained growth [3, 4]. Among those targets, chemokines have long been associated with cancer progression, as various chemokines provide a supportive environment for tumor cells, like “an inflammatory wound that never heals” [5]. Compared with other chemokines, CXCL-8 (also called interleukin 8, or IL-8) of the CXC chemokine family deserves more attention as a player that regulates many cellular functions of melanoma.

CXCL-8, hardly detectable in normal cells, is constitutively secreted by melanoma cells [6, 7]. Two high-affinity receptors for CXCL-8, CXCR1 and CXCR2, are differentially expressed on melanoma and endothelial cells [8, 9]. Upon activation of CXCL-8 signaling, various downstream pathways and transcription factors involved in cell proliferation, apoptosis suppression, cell cycle control, and cytoskeletal dynamics are turned on [10]. Our previous studies found a positive correlation between CXCL-8 and its receptor expression and melanoma aggressiveness [9]. CXCL-8, induced by factors such as ultraviolet B irradiation, contributes to increased melanoma burden in nude mice [11]. Inhibition of CXCL-8 signaling or its receptors shows therapeutic efficacy against melanoma in preclinical studies [12, 13].

In various other cancers, including lung cancer, prostate cancer, and squamous cell carcinoma, a similar tumor-promoting role of CXCL-8 signaling has been found [10, 14, 15]. Notably, a recent study characterized expression of the CXCL-8 receptor CXCR1 on breast cancer stem cells, which we believe to be the most aggressive tumor subpopulation, and described CXCR1 blockade decreases the docetaxel-amplified cancer stem cell population [16]. In this study, we evaluated the critical role of CXCL-8 in melanoma growth and progression by modulating its expression in melanoma cell lines expressing different levels of CXCL-8. We observed that the CXCL-8 level is a direct indicator of multiple tumor behaviors, including growth, angiogenesis, and metastasis.

## Material and Methods

### Construction of plasmid expressing CXCL-8 antisense RNA

Plasmid (BCMGS/neo) carrying CXCL-8 complementary DNA (cDNA) was a kind gift from Dr. K. Matsushima (Kanazawa University, Kanazawa, Japan). For construction of plasmid containing CXCL-8 antisense RNA, CXCL-8 cDNA fragment was excised from BCMGS/CXCL-8 using *XhoI–NotI* restriction enzymes. The fragment obtained after digestion was cloned in an antisense orientation at *NotI–XhoI* restriction site in pcDNA/neo vector from Invitrogen (Carlsbad, CA). The insert sequence and orientation were confirmed by sequencing the clones.

### Cell culture and stable transfection

The human melanoma cell lines A375P (medium metastatic) and A375SM (highly metastatic) were maintained in culture as an adherent monolayer in Dulbecco's Modified Eagle's Medium (DMEM) (MediaTech, Herndon, VA), supplemented with 5% fetal bovine serum (FBS), 1% l-glutamine, 1% vitamin solution, and gentamycin. A375P and A375SM cells (5 × 10^5^ cells/dish) grown in 100-mm culture dishes (at 60–80% confluence) were transfected with BCMGS/neo or pcDNA3.1/neo for control (A375P control or A375SM control) and with BCMGS/CXCL-8 or pcDNA3.1/CXCL-8 antisense plasmids for modulation of CXCL-8 in the cells (A375P-CXCL-8 or A375SM-anti-CXCL-8) using Lipofectamine (Invitrogen) according to the manufacturer's instructions. The cells were switched to a selective medium containing Geneticin (G418; 800–1000 *μ*g/mL; Invitrogen) 48 h following transfection and G418-resistant pooled populations were obtained and used.

### RNA isolation and northern blot analysis

Total RNA from in vitro cultured cells was isolated using Trizol® reagent (Invitrogen), and northern blot analysis was performed using cDNA probes as described in our previous study [17]. CXCL-8 mRNA expression was quantitated using Phosphor imager and ImageQuant software (Molecular Dynamics, Sunnyvale, CA).

### Enzyme-linked immunosorbent assay

Cell-free culture supernatants and serum samples were analyzed for CXCL-8 protein levels using enzyme-linked immunosorbent assay (ELISA) paired antibody assay kit (R&D Systems Inc., Minneapolis, MN) according to manufacturer's instructions.

### Cell proliferation assay

Cell proliferation was determined by MTT (3-[4,5-dimethylthiazol-2-yl]-2,5-diphenyltetrazolium bromide, a tetrazole) assay as previously described [17–19]. Growth was calculated as percent (%) = [{(A/B) − 1} × 100], where A and B are the absorbance of transfected cells and control cells, respectively.

### Cell motility and invasion assay

To investigate the effect of CXCL-8 modulation on melanoma cell invasiveness, in vitro cell motility and invasion assay was performed as described earlier [18, 19]. Migrated cells were stained using Hema 3 kit (Fisher Scientific Company L.L.C., Kalamazoo, MI) using manufacturer's instructions and counted in 10 random fields (200×) using Nikon microscope.

### Soft-agar colony formation assay

To analyze growth rates of cells stably transfected with sense CXCL-8, antisense CXCL-8, or vector control in soft agar, six-well plates were coated with 1.5-mL base agar of different concentrations (0.3–1.2%), DMEM, and 5% FBS. Cells were trypsinized and counted; 5 × 10^3^ cells were suspended in 0.3–1.2% low-melting agarose and then layered on top of the base agar in six-well culture plates. Cells were incubated at 37°C in a humidified incubator for 2 weeks. The plates were stained with 0.5 mL of 0.005% crystal violet in methanol and colonies were counted under a dissecting microscope.

### In vivo tumor growth, spontaneous and experimental lung metastasis

Female athymic nude (6- to 8-week-old) were purchased from the National Cancer Institute and used according to procedures approved by the University of Nebraska Medical Center Institutional Animal Care and Use Committee as described earlier. A375SM-control, A375SM-anti-CXCL-8, A375P-control, or A375P-CXCL-8 cells (1 × 10^6^ cells/0.1 mL of HBSS [Hank's Balanced Salt Solution]) were injected subcutaneously (s.c.) and tumor growth was monitored. For spontaneous metastasis, primary tumors were removed and animals were monitored for another 8 weeks.

Tumor volume was calculated using the formula π/6 × (smaller diameter)^2^ × (larger diameter) as described earlier [18, 19]. Tumors were fixed in zinc fixative and processed for histopathological evaluation. To examine spontaneous lung metastasis, mice were killed 8 weeks following primary tumor removal and their lungs were examined for metastases.

For experimental lung metastasis, A375P- and A375SM-transfected cells (1 × 10^6^ cells/0.1 mL of HBSS) were injected intravenously (i.v.) and mice were sacrificed 8 weeks later. Harvested lungs were fixed in Bouin's solution and metastatic nodules were counted under a dissecting microscope.

### Immunohistochemistry

Immunohistochemical analysis was performed as previously described [7]. The following primary antibodies were used: anti-proliferating cell nuclear antigen (PCNA) (1:40; Santa Cruz Biotechnology, Santa Cruz, CA), anti-CXCL-8 (1:200; Endogen, Woburn, MA), and biotinylated GS-IB4 (1:50; isolectinB4 from Griffonia simplicifolia; Vector Laboratories, Burlingame, CA). Immunoreactivity was visualized by incubation with avidin–biotin complex and diaminobenzidine tetrahydrochloride substrate (Vector Laboratories). TUNEL apoptosis assay was performed using DeadEnd colorimetric TUNEL assay system (Promega, Madison, Wisconsin) using manufacturer's protocol. Immunostained cells and microvessels were counted microscopically (Nikon E400 microscope) using a 5 × 5 reticle grid.

### Statistical analysis

In vitro analysis was performed using unpaired two-tailed *t*-test using SPSS software (SPSS Inc., Chicago, IL). In vivo analysis was done using the Mann–Whitney *U*-test. All the values were expressed as mean ± SEM. Bivariate correlation analysis was performed using Spearman's rho correlation coefficient for nonparametric distributions. Correlation coefficient ranges from −1 (a perfect negative relationship) to +1 (a perfect positive relationship). When interpreting these results, no cause–effect conclusions are made as a result of significant correlations. A *P*-value of equal or less than 0.05 was considered statistically significant.

## Results

### Clonogenic ability and proliferative potential of melanoma cells with different levels of CXCL-8 expression

**Figure 1:**
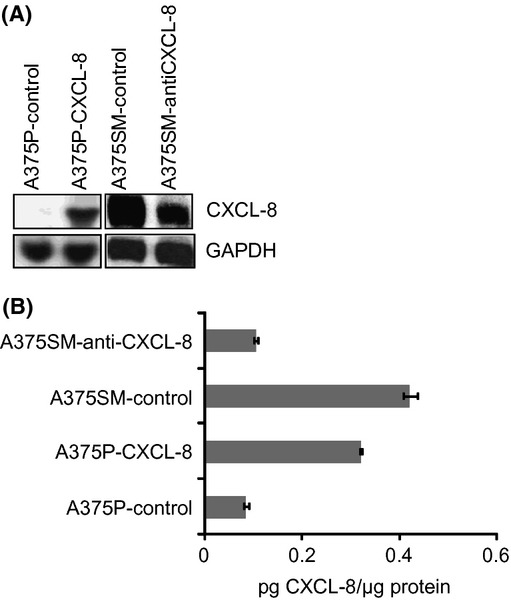
CXCL-8 overexpression and knockdown in A375P and A375SM melanoma cells. (A) Northern blot analysis to confirm CXCL-8 mRNA expression in A375P-control, A375P-CXCL-8, A375SM-control, and A375SM-anti-CXCL-8 cells. GAPDH was used as control. (B) CXCL-8 protein secretion in CXCL-8 overexpressing and knockdown cells as determined using ELISA and presented as mean CXCL-8 levels in pg/*μ*g of total protein ± SEM.

Pooled sublines were selected after growing the cells in G418-containing media. The insertion, orientation, and expression of inserts were determined by reverse transcription polymerase chain reaction (RT-PCR; data not shown). CXCL-8 expression in the derived sublines was examined using northern blot (Fig. 1A) and ELISA (Fig. 1B) analysis. The selected sublines A375P-control, A375P-CXCL-8, A375SM-control, and A375SM-anti-CXCL-8 were monitored over a period of 1–2 months for stable and consistent expression of CXCL-8.

Two weeks after plating the cells in soft agar, we evaluated the number of colonies formed for each cell line. A375P-CXCL-8 cells exhibited higher clonogenic ability as compared with control cells (Table 1 – upper panel). On the other hand, when knockdown cells were plated on soft agar, they exhibited a decrease in clonogenic ability as compared with control cells (Table 1 – lower panel). Regarding clonogenic ability, all the sublines were better at a lower concentration of agarose. No obvious differences in the morphology of individual colonies were observed.

**Table 1:** CXCL-8 expression in melanoma cells modulates clonogenic potential in soft agarose

	Agarose concentration in top layer (%)			
	
Cell lines	0.3	0.6	0.9	1.2
A375P-control	0 ± 0	0	0	0
A375P-CXCL-8	12.66^1^ ± 4.16*	9.33 ± 2.52*	7.3 ± 2.5*	0

A375SM-control	132 ± 31	87 ± 25	57 ± 17	32 ± 8
A375SM-anti-CXCL-8	56 ± 4*	38 ± 2*	24 ± 5*	9 ± 3*

Cells were plated in agarose (0.3–1.2% v/v) and incubated for 14 days. Colonies were counted using an inverted microscope. The values are mean number of colonies ± SEM of duplicate cultures. This is one representative of three experiments done in duplicate.

* Significant difference from control cell lines (*P* < 0.05).

**Figure 2:**
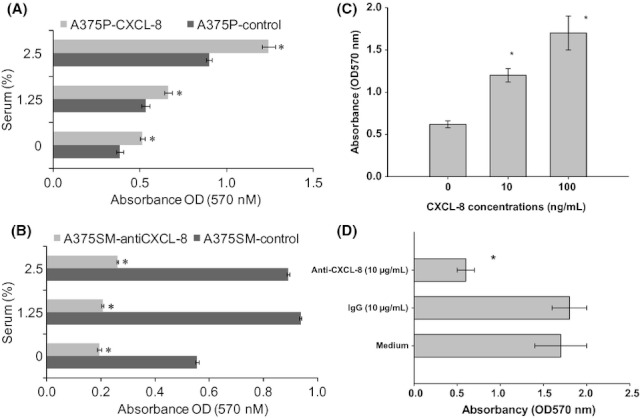
Modulation of cell proliferation following altered CXCL-8 expression in melanoma cells. (A) and (B) In vitro cell proliferation of A375P-control, A375P-CXCL-8, A375SM-control, and A375SM-anti-CXCL-8 cells was determined at 72 h by MTT assay. (C) A375P cells were treated with media alone or media containing different concentrations of CXCL-8. (D) A375SM cells were incubated with media alone, media containing control IgG or neutralizing anti-CXCL-8 antibody. Cell proliferation was determined using MTT assay and presented as mean absorbance OD_570nm_ ± SEM. *Significantly different from controls (*P* < 0.05).

To determine growth potential of transfected cells, in vitro cell proliferation was examined by MTT assay. Cells were seeded at low density (1000 cells/well) into 96-well plates in medium alone or medium containing different concentrations of serum (0–2.5%). As reported in Figure 2A, overexpression of CXCL-8 enhances cell proliferation (33–38%) in A375P-CXCL-8 cells as compared with A375P-control cells, whereas knockdown cells showed a 65–78% decrease in proliferation as compared with their respective control (Fig. 2B).

Furthermore, we treated A375P and A375SM cells expressing different levels of CXCL-8 with exogenous CXCL8 or neutralizing CXCL8 antibody. We observed significant increase in A375P cell proliferation following addition of exogenous CXCL8 in the culture medium (Fig. 2C). Neutralization of CXCL8 activity in A375SM cells inhibited its proliferation (Fig. 2D). Taken together, our data show that modulation of CXCL-8 levels affects the clonogenic and growth potential of melanoma cells.

### CXCL-8 expression affects motility and invasion of melanoma cells

**Figure 3:**
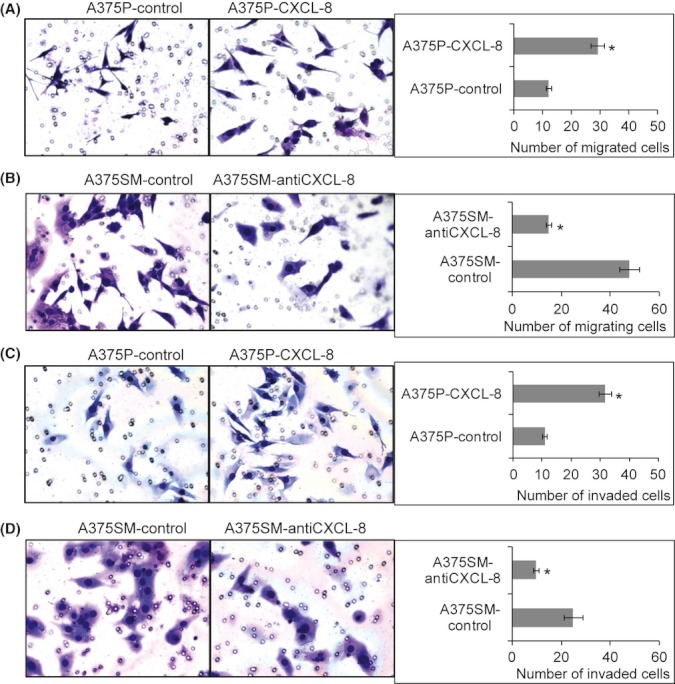
Modulation of CXCL-8 expression regulates cell motility and invasion. A375P-control, A375P-CXCL-8, A375SM-control, and A375SM-anti-CXCL-8 cells were seeded on noncoated (cell motility, A and B) or Matrigel-coated (cell invasion, C and D) membranes for migration assays. Migrated (A and B) and invaded (C and D) cells were counted and presented as average number of cells/field ± SEM (a representative of three experiments done in triplicate). *Significantly different from controls (*P* < 0.05).

To understand the role of CXCL-8 in tumor progression, we used transwell assays to analyze migration through a porous membrane toward serum (chemotaxis) for cells with different levels of CXCL-8. Increased migration (2.4-fold) was observed when A375P cells overexpressed CXCL-8, while A375SM cells downregulated for CXCL-8 showed decreased (3.2-fold) migration (Fig. 3A and B). We further examined whether cell motility was also associated with CXCL-8 levels in these cells. Invasiveness of the melanoma cells was determined by invasion of the cells through Matrigel in an in vitro assay. Our results showed an increased number (2.9-fold) of invading A375P-CXCL-8 cells, while there was a decreased number (2.6-fold) of invading A375SM-anti-CXCL-8 cells as compared with controls (Fig. 3C and D).

### Modulation of CXCL-8 expression affects melanoma growth

**Figure 4:**
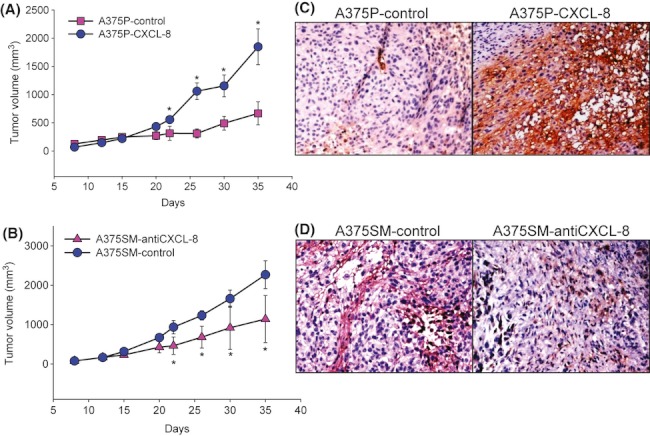
Modulation of CXCL-8 expression alters melanoma growth in vivo. Melanoma cells (A375P-control, A375P-CXCL-8, A375SM-control, and A375SM-anti-CXCL-8) were injected s.c. and tumor volume was monitored. (A) Growth of A375P-control and A375P-CXCL-8 tumors. (B) Growth of A375SM-control and A375SM-anti-CXCL-8 tumors. (C) and (D) Immunohistochemical staining for CXCL-8 in A375P-control, A375P-CXCL-8, A375SM-control, and A375SM-anti-CXCL-8 tumors to show differential levels of CXCL-8. *Significantly different from controls (*P* < 0.05).

A375P- and A375SM-derived heterogeneous populations were used to study the effect of CXCL-8 on tumor cell growth in immunodeficient mice. A total of five immunodeficient mice per group were injected, and tumors were removed on day 35 after tumor implantation. All injected mice (A375P-control, A375P-CXCL-8, A375SM-control, and A375SM-anti-CXCL-8) showed a palpable tumor within 10-day postinjection, tumor volume was recorded and tumors were resected on day 35. A significant increase (2.7-fold) in tumor volume was observed in the A375P-CXCL-8 group, as compared with the A375P-control group on day 35 (Fig. 4A). In contrast, a significant decrease in tumor growth (2.0-fold) was observed in the A375SM-anti-CXCL-8 group as compared with the A375SM-control group (Fig. 4B). Immunohistochemical analysis of the primary tumor sections was done using an anti-CXCL-8 mouse monoclonal antibody to evaluate the expression of CXCL-8. Staining confirmed sustained expression of CXCL-8 in tumors derived from A375P-control, A375P-CXCL-8, A375SM-control, and A375SM-anti-CXCL-8 cells (Fig. 4C and D).

### CXCL-8 expression regulates spontaneous and experimental melanoma metastasis

The incidence of spontaneous metastasis in A375P-CXCL-8 cells in nude mice was 100% as compared with 30% in mice injected with A375P-control cells (Table 2). The number of lung nodules in A375P-CXCL-8-injected mice ranged from 1 to 8 (median 3) as compared with 0 to 3 (median 0) in A375P-control. In contrast, the incidence of spontaneous lung metastasis of highly metastatic A375SM melanoma cells was greatly inhibited when CXCL-8 was downregulated (Table 2). A375SM-anti-CXCL-8 cells resulted in very few lung nodules ranging from 0 to 4 (median 0), as compared with 3 to 11 (median 5) in the A375SM-control group.

**Table 2:** CXCL-8 expression regulates spontaneous and experimental melanoma metastasis

Cell lines	Spontaneous metastasis incidence (%)	Lung metastasis median (range)	Experimental metastasis incidence (%)	Lung metastasis median (range)
A375P control	30	0 (0–3)	100	20 (2–50)
A375P-CXCL-8	100*	3 (1–8)	100	60 (41–110)
A375SM control	80	5 (3–11)	100	100 (30–250)
A375SM-anti-CXCL-8	20*	0 (0–4)	100	8 (1–24)

Cells were injected into BALB/c nude mice (*n* = 5) for spontaneous and experimental lung metastasis as described in Material and Methods. Incidence was calculated by comparing number of mice with metastases with total number of mice injected. Median number of lung nodules has been shown with range.

* Significant difference from control tumor-bearing animals (*P* < 0.05).

Regardless of the cell line used for i.v. injection, all the mice had 100% experimental metastasis incidence, but there was a difference in the number of lung nodules (Table 2). The number of lung nodules in A375P-CXCL-8-injected mice ranged from 41 to 110 (median 60) as compared with 2 to 50 (median 20) in A375P control. The number of lung nodules in A375SM-anti-CXCL-8-injected mice ranged from 1 to 24 (median 8) as compared with 30 to 250 (median 100) in A375SM control.

### Melanoma CXCL-8 expression modulates tumor neovascularization

**Figure 5:**
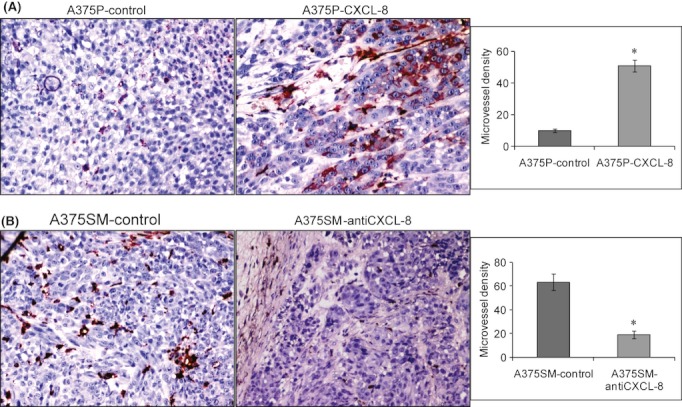
Melanoma CXCL-8 expression modulates tumor neovascularization. A375P-control, A375P-CXCL-8, A375SM-control, and A375SM-anti-CXCL-8 tumors were immunostained for microvessel density. (A) A375P-CXCL-8 tumors show higher immunostaining as compared with A375P-control tumors. (B) A decrease in microvessel density in A375SM-anti-CXCL-8 tumors as compared with A375SM-control tumors. The representative pictures are shown at 200×. Quantitation of microvessel density in tumors with different CXCL-8 levels was made using a 5 × 5 reticle grid at 400× magnifications and presented as average number of microvessels ± SEM. *Significantly different from controls (*P* < 0.05).

Having confirmed the effect of CXCL-8 on tumor growth, we next examined its role in neovascularization in vivo. The number of blood vessels was compared in tumors by immunohistochemical staining using biotinylated isolectin B4 (GS-IB4). The average number of blood vessels was counted, excluding necrotic areas. Staining in A375P-CXCL-8 tumor sections showed a 5.0-fold increase in the number of blood vessels as compared with A375P-control tumors (Fig. 5A). In contrast, in A375SM-anti-CXCL-8 tumors, we observed a 3.3-fold decrease in the number of tumor blood vessels as compared with A375SM-control tumors (Fig. 5B).

### Modulation of CXCL-8 expression alters in situ cell proliferation and survival

**Figure 6:**
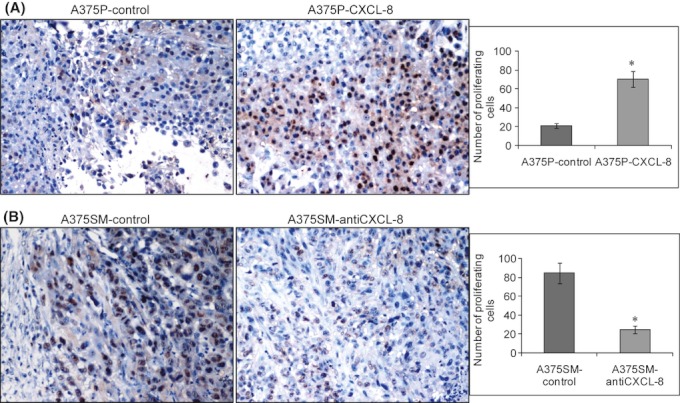
Altered melanoma cell proliferation in vivo in melanoma tumors expressing different levels of CXCL-8. PCNA immunohistochemical staining was performed, and the number of proliferating cells in A375P-control and A375P-CXCL-8 tumors (A) and in A375SM-control and A375SM-anti-CXCL-8 tumors (B) was counted and presented as average number of cells/field ± SEM (right panel). *Significantly different from controls (*P* < 0.05).

**Figure 7:**
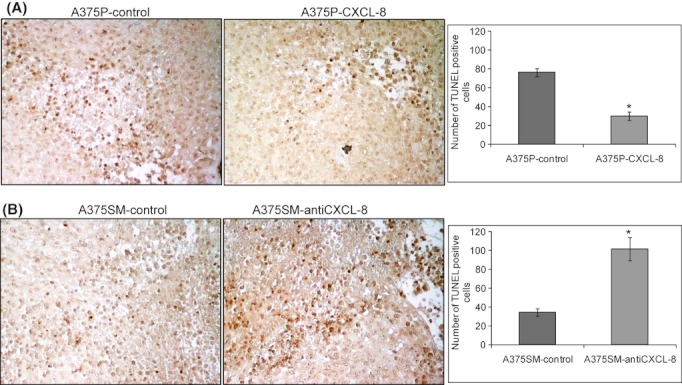
Frequency of apoptotic melanoma cells in vivo in melanoma tumors expressing different levels of CXCL-8. TUNEL staining was performed in the tumor section. The number of apoptotic cells in A375P-control and A375P-CXCL-8 tumors (A), and A375SM-control and A375SM-anti-CXCL-8 tumors (B) was counted and presented as average number of cells/field ± SEM (right panel). *Significantly different from controls (*P* < 0.05).

Because the overall rate of cell growth is determined by the balance between cell proliferation and apoptosis, we next determined the proliferation and apoptotic indices of A375P- and A375SM-derived tumors. We counted nuclear PCNA-positive cells in different tumors. The average number of PCNA-positive cells by immunohistochemical staining showed an increase for A375P-CXCL-8 tumors (3.3-fold) compared with A375P-control tumors (Fig. 6A). A decrease of 3.4-fold for A375SM-anti-CXCL-8 tumors as compared with A375SM-control tumors was observed (Fig. 6B). TUNEL analysis of cell death showed a significant decrease of 2.5-fold (A375P-CXCL-8) as compared with control tumors (Fig. 7A). In contrast, A375SM-anti-CXCL-8 tumors had significantly increased numbers of TUNEL-positive cells as compared with A375SM-control tumors. The number of apoptotic cells in A375SM-anti-CXCL-8 tumors was 3.0-fold increased over control tumors (Fig. 7B).

### Serum CXCL-8 levels correlate with tumor burden and spontaneous lung metastasis

**Figure 8:**
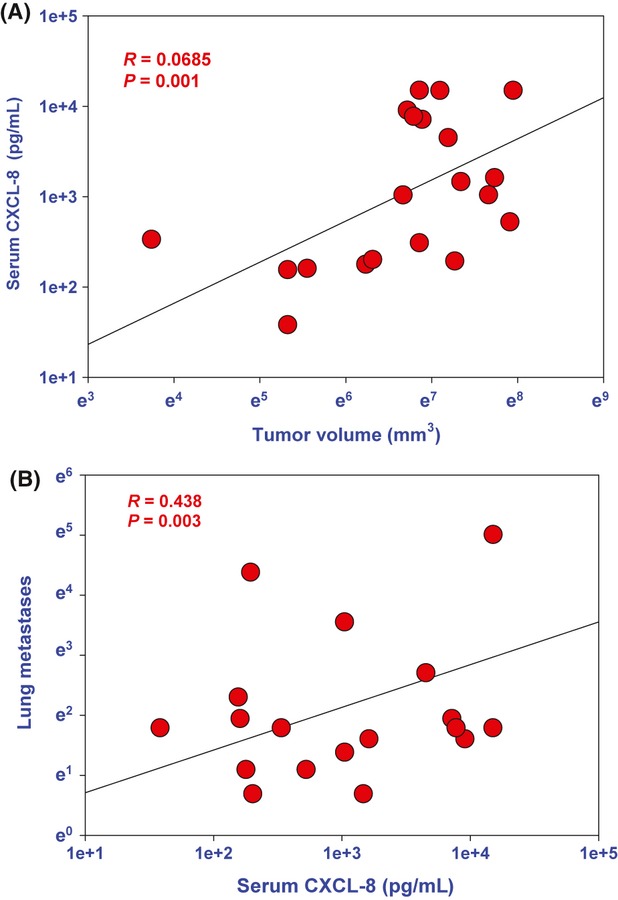
Serum CXCL-8 level correlates with tumor burden and spontaneous lung metastasis. Serum from tumor-bearing animals was collected at the time of primary tumor removal, and CXCL-8 levels were determined using ELISA. The values are mean CXCL-8 levels (in pg/mL) ± SEM. *Significantly different from controls (*P* < 0.05).

In order to examine the preclinical prognostic significance of CXCL-8 level in melanoma growth and metastasis, we examined the serum CXCL-8 levels and determined whether there is any correlation between them. Serum samples were collected at the time of primary tumor removal. We observed significant correlation between serum CXCL-8 level and melanoma burden (Fig. 8A) and spontaneous metastasis (Fig. 8B), demonstrating serum CXCL-8 as an important preclinical diagnostic marker.

## Discussion

More than two decades of research have strongly documented the fact that chemokines and chemokine receptors are often strongly upregulated during tumorigenesis, which can promote growth, increase angiogenesis, and facilitate metastasis [20, 21]. Even though chemokines have gained attention, the knowledge regarding the direct involvement of CXCL-8 in melanoma progression is not completely understood. Our recent studies have demonstrated that CXCR1 and CXCR2, receptors of CXCL-8, are functionally involved in melanoma progression [13, 19]. In addition, we have also shown that host CXCR2 plays a critical role in melanoma growth, angiogenesis, and experimental metastasis [22]. Interestingly, different chemokine receptors direct tumor cells to specific organs in a mouse model, for example, CCR7, CCR10, and CXCR4 to lymph node, skin, and lung metastasis, respectively [23]. Chemokines can be autocrine or paracrine growth factors for melanoma tumors. For example, CXCL1-3, CXCL-8, and CCL-5 have been reported to favor melanoma growth and/or progression. Specifically, CXCL1-3 expression accounts for enhanced tumorigenicity in nude mice via malignant transformation of melanocytes as well as enhanced microvessel growth to support tumor progression [24–26]. CCL-5-expressing melanoma cells generate tumors in mice, and the tumor burden is correlated with CCL-5 levels [27]. CCL-5 might provide a supporting environment for tumor growth by recruiting leukocytes, such as monocytes and T cells to the tumor.

In this study, we evaluated the direct involvement of CXCL-8 in melanoma progression. We established two cell line models first by enhancing CXCL-8 expression in the medium metastatic A375P cells which produce low levels of CXCL-8, and next lowering CXCL-8 in the highly metastatic A375SM cells which produce high levels of CXCL-8, thus generating A375P-CXCL-8 and A375SM-anti-CXCL-8. Our observations were increased tumor volumes in A375P-CXCL-8-injected mice, whereas tumor volumes in A375SM-anti-CXCL-8-injected mice were decreased, compared with controls, which confirmed the role of CXCL-8 in melanoma cell growth. More important, we demonstrated that serum CXCL-8 level correlates with tumor burden and spontaneous lung metastasis in melanoma-bearing mice. These results strengthen our previous report that modulation of CXCL-8 expression in melanoma cells enhances tumor growth and metastasis [6, 17].

Our observation is in agreement with our cell proliferation and survival data from the A375P- and A375SM-derived sublines. We observed enhanced cell proliferation in A375P-CXCL-8 tumors compared with A375P-control tumors, and the opposite when CXCL-8 was suppressed in A375SM-anti-CXCL-8 tumors. This might be explained by the activation or suppression of cellular pathways downstream of CXCL-8 signaling. Among them, the mitogen-activated protein kinase (MAPK) pathway is constitutively activated in melanoma by mutations or exogenous stimulation of growth factors [28]. Our previous studies have shown that exogenous CXCL-8 stimulated extracellular signal-regulated kinases (ERK) phosphorylation in melanoma cell lines, which is involved in CXCR1- or CXCR2-mediated cell growth [18]. In addition to changes in cell proliferation, we observed decreased cell death by TUNEL analysis in A375P-CXCL-8 tumors as compared with control tumors, and increased apoptosis in A375SM-anti-CXCL-8 tumors versus control. The role of CXCL-8 in regulating antiapoptotic genes in melanoma has not been studied here, but it has been reported in androgen-independent prostate cancer cells, where Bcl-2 and inhibitors of apoptosis (IAP) family gene transcripts were upregulated via NF-κB activation stimulated by CXCL-8, thus rendering tumor cells more resistant to the cytotoxic oxaliplatin treatment [29].

New tumor blood vessels are recruited and formed to facilitate tumor growth beyond a certain size, supporting further tumor invasion and metastasis. In our in vivo studies, a significant increase in blood vessel number and density was found in A375P- and A375SM-derived tumor sections with higher CXCL-8 levels, as compared with their counterparts. Tumor angiogenesis occurs upon stimulation of angiogenic growth factors. Our previous work has characterized the role of CXCL-8 as an angiogenic signal for endothelial cells, which express its receptors CXCR1 and CXCR2 [18, 30]. In addition to CXCL-8-favored endothelial cell proliferation and antiapoptotic effects due to enhanced transcription of Bcl-2 and Bcl-x_L_, there was increased endothelial cell migration toward the angiogenic signal, which is a critical step for sprouting vessels to extend. Proteases are released to facilitate migrating endothelial cell degradation of the basement membrane, and the CXCL-8-activated matrix metalloproteinase-2 (MMP-2) and MMP-9 observed in those studies are among the potential contributing proteases [30, 31]. The multiple CXCL-8-regulated biological activities in endothelial cells provide an underlying mechanism for our current findings that modulation of CXCL-8 levels affects melanoma angiogenesis.

Similarly, CXCL-8 regulates the process of melanoma cell local migration and distant metastasis. The incidence of spontaneous metastasis and number of metastatic lung nodules were increased in A375P-CXCL-8-injected mice but greatly inhibited in A375SM-anti-CXCL-8-injected mice, compared with A375P- and A375SM-control injected mice. Comparison of lung metastasis in mice in the experimental metastasis models implanted with A375P- and A375SM-derived tumor cells showed similar results. This is in line with our in vitro analysis of cell migration and invasive potential. MMPs might also be an important player here. We did not examine the potential change in MMP activity in our CXCL-8 overexpressing or knocked-down cells, but Luca et al. reported increased MMP-2 activity that correlated with metastatic potential in CXCL-8-transfected SB-2 cells [32]. Another study in androgen-independent prostate cancer described a similar role for CXCL-8 induction of MMP-9 in tumor invasion and tumor-induced neovascularization [33]. Therefore, CXCL-8 potentially promotes the transcription of various extracellular matrix degrading enzymes in specific cancer cells, which facilitates their escape from primary sites.

Using different cell models, we confirmed similar observations from Luca et al. [32] and Inoue et al. [33], where they enforced CXCL-8 expression in poorly tumorigenic melanoma or prostate cancer cell lines with low levels of CXCL-8, and reported enhanced tumor aggressiveness. Conversely, antisense transfection of CXCL-8 in cancer cells inhibited multiple tumor activities. In this study, we modulated CXCL-8 levels to mimic the gain or loss of CXCL-8 function in melanoma cell lines. On the one hand, CXCL-8 can be stimulated by a stress environment or pre-inflammatory cytokines to rescue cell death or promote tumor progression. For example, dacarbazine, the standard therapy for melanoma, causes a “chemokine storm” of CXCL-8 [34] and other CXC chemokines of the same receptor signaling after massive cell death (unpublished data from our laboratory), which is one of the escape mechanisms for developing therapy resistance. Tumor hypoxia upregulated CXCL-8 production is also credited for increased tumor dissemination and aggressiveness [35, 36]. On the other hand, suppressing the CXCL-8-dependent pathway provides a valid rationale for melanoma therapy.

In summary, our results suggest CXCL-8 is a direct determinant of aggressive melanoma phenotypes, including tumor growth, metastasis, and angiogenesis, and targeting CXCL-8 produced by tumor cells and the supporting stroma is a direction for studying this pathway to develop future melanoma diagnosis and therapies.
